# Hyperphosphorylated FAK Delocalizes from Focal Adhesions to Membrane Ruffles

**DOI:** 10.1155/2010/932803

**Published:** 2010-08-19

**Authors:** Abdelkader Hamadi, Therese B. Deramaudt, Kenneth Takeda, Philippe Rondé

**Affiliations:** ^1^Laboratoire de Biophotonique et Pharmacologie, CNRS, UMR 7213, 74 rte du Rhin, 67401 Illkirch, France; ^2^Faculté de Pharmacie, Université de Strasbourg, 67401 Illkirch, France

## Abstract

Cell adhesion and migration are key determinants in tumor metastasis. Adherence of tumor cell to the extracellular matrix is mediated via integrin containing focal adhesions (FAs). Binding of integrins to ECM triggers phosphorylation of two major components of FAs, focal adhesion kinase (FAK) and Src, activating downstream signaling pathway which leads to FA disassembly and cell migration. In this paper, we analyze how phosphorylation of FAK regulates its trafficking at FAs in living human astrocytoma cells. Upon pervanadate-induced FAK phosphorylation, phosphorylated FAK appeared highly expressed at newly formed membrane ruffles. This effect was abolished in presence of the specific Src inhibitor PP2. Our findings demonstrate that upon phosphorylation, FAK delocalizes from FAs to membrane ruffles.

## 1. Introduction

During the process of tumor metastasis and more generally cell migration, cells connect with the microenvironment in part through focal adhesions (FAs) [1]. FAs transduce signals from the extracellular matrix into the cell via integrins clustering and subsequent activation of signaling pathways. One of the major kinases implicated in FA signaling is focal adhesion kinase (FAK). FAK contributes to FA scaffolding, and also transmits adhesion-dependent and growth factor-dependent signals into the cell [2–4]. A major role of FAK is to influence the dynamic regulation of integrin-associated adhesions, and the actin cytoskeleton that is tethered there, through diverse molecular interactions. This in turn regulates cell migration by controlling the focal complex assembly/disassembly cycle at the leading lamellipodia of migrating cells, while also controlling adhesion disassembly at the trailing edge.

As FAK^−/−^ fibroblasts show excessive, rather than decreased, formation of focal contacts, FAK has been associated with the disassembly of integrin-based adhesion sites [5]. Indeed, the apparent rate constants for incorporation into FAs of paxillin and zyxin, two FA components, were similar to those observed in wild-type fibroblasts, indicating that the rate of FA formation is not affected by the absence of FAK [6]. On the other hand, the FA disassembly in FAK^−/−^ cells was significantly impaired, as the rate constants for the disassembly of paxillin and zyxin were about 14-fold less in FAK^−/−^ cells compared to wild-type cells. When FAK was reintroduced in FAK^−/−^ cells, the rate constant for paxillin disassembly was comparable to that observed for wild-type cells [6]. Consistent with the proposal that FAK modulates adhesion turnover, the number of adhesions that turned over in protrusive regions of FAK^−/−^ cells was markedly less than in wild-type cells. Taken together, these results show that FAK is necessary for the efficient disassembly of dynamic adhesions.

Tyr397 is a major autophosphorylation site in FAK and phosphorylation at Tyr397 creates a binding site for Src family kinases and Src homology 2-containing proteins, suggesting a role of Src in adhesion turnover regulation [3]. Hence, the apparent rate constant for paxillin disassembly decreased 19-fold in Src/Yes/Fyn-deficient fibroblasts, as compared to wild-type fibroblasts. Comparable results were observed in FAK^−/−^ cells expressing Y397F-FAK [6]. Thus, a common signaling pathway leading to FA disassembly appears to require a phosphorylation step. In agreement, pervanadate-induced hyperphosphorylation of FAK resulted in the exclusion of FAK from FAs [7]. Moreover, ERK/MAP kinase phosphorylation is necessary for calpain2 activation which leads to FA turnover, with the formation of the active complex consisting of at least ERK/MAP kinase and calpain2 being driven by the adaptor function of FAK [8]. Several other studies have shown that phosphorylation of FAK is associated with FA disassembly and thus regulates cell migration [9–12]. We previously reported the existence of a rapid flux of FAK between cytosolic and FA compartments in U87 astrocytoma cells, as revealed by FRAP analysis [13]. Furthermore, phosphorylation of FAK at Tyr397 increases specifically the time-residency of FAK at FAs but not in cytosol, which in turn induces disassembly of FAs as observed using Y397F-FAK mutant cells [14]. In order to uncover the relationship between FAK trafficking and phosphorylation events, we analyzed both the level of FAK phosphorylation upon pervanadate treatment in different cell compartments and FAK dynamics in living cells. Using this approach, we demonstrated that increase in FAK phosphorylation induces delocalization of FAK from FAs to membrane ruffles. This effect was mediated by Src as shown using both selective inhibitor of Src kinase and overexpression of the kinase dead mutant of Src (K298M-Src).

## 2. Materials and Methods

### 2.1. Reagents and Antibodies

Eagle's minimum essential medium (EMEM), fetal bovine serum (FBS), ultraglutamine, penicillin, streptomycin, and trypsin-EDTA solution were from Lonza. Matrigel and mouse monoclonal antibody (Ab) directed against FAK kinase domain (amino acids 354–533) were from BD Biosciences. Polyclonal Abs directed against the C-terminal (Ct) domain of FAK (amino acids 748–1052) was from Upstate. Antiphospho-Tyr861-FAK (p-Tyr861) and antiphospho-Tyr397-FAK (p-Tyr397) Abs were from BioSource. Anti-Src Ab was from Biomol International. Anticortactin was from Millipore. Antivinculin and *β*-actin Abs were from Sigma. Phalloidin fluoroProbe547 was from Interchim. Horseradish peroxidase-conjugated goat antimouse or antirabbit IgG were from Promega. Rhodamine Red X-conjugated goat antimouse or anti-rabbit Abs were from Jackson Laboratories. Protease inhibitor mixture tablets and Fugene 6 were obtained from Roche. PP2 and G418 sulfate were from Invitrogen. Pervanadate solution at 1 mM was freshly prepared by mixing vanadate solution (from Sigma) and hydrogen peroxide in PBS to final concentrations of 1 mM and 0.006%, respectively. A final concentration of 100 *μ*M pervanadate was used to treat the cells.

### 2.2. Expression Vectors

pcDNA3-FAK/YCam and Y397F-FAK/YCam were constructed as described previously [14]. The pcDNA3 plasmid containing Y530F-Src was kindly provided by F. Cruzalegui. All plasmids were isolated using the JetStar Plasmid kit (Genomed) following the manufacturer's protocol.

### 2.3. Cell Culture and Stable Transfections

U87-MG human astrocytoma cell line was obtained from the American Type Culture Collection. Cells were maintained as subconfluent monolayers in EMEM supplemented with 10% FBS, 2 mM ultraglutamine, 100 units/ml penicillin, and 100 *μ*g/ml streptomycin. For stable transfection, cells were first transfected using Fugene 6, transfected cells were selected by fluorescence-activated cell sorting and maintained in 1 mg/ml G418 containing medium.

### 2.4. Immunoblotting

For western blots, FAK/YCam-expressed cells were plated at low density on dishes precoated with 178 *μ*g/ml Matrigel for 2 days. Cells were then washed with cold PBS and lysed with ice-cold RIPA buffer (150 mM NaCl, 1% Triton X-100, 0.5% Na deoxycholate, 0.1% SDS, 50 mM Tris-HCl, pH 7.5, and a protease inhibitor mixture tablet). Protein lysates were resolved by SDS-PAGE and then transferred to polyvinylidene difluoride membrane (GE healthcare). After 1 h blocking at room temperature in 0.1% casein-PBST (PBS supplemented with 0.1% Tween 20), membranes were incubated overnight with primary Abs at 4°C: anti-FAK kinase (1/1000), anti-FAK Ct (1/1000), antiphospho FAK (Tyr397 or Tyr861 at 1/2000). Corresponding horseradish peroxidase-conjugated secondary Abs were used at 1/30000 dilution. Immunoreactivity was visualized using the ECL+ system (GE healthcare).

### 2.5. Indirect Immunofluorescence and Correlation Analysis

FAK/YCam-expressing cells were plated at low density on Matrigel for 2 days before paraformaldehyde fixation, 0.2% Triton X-100 and blocking with PBS/3% BSA. Cells were washed with PBS and incubated with primary Abs (1/200) in PBS/0.2% BSA for 1 h. After additional washes, cells were incubated with rhodamine Red X-conjugated secondary Abs in PBS/0.2% BSA (1/200), washed with PBS and then observed using a confocal microscope (Bio-Rad 1024, Kr-Ar laser; Nikon Eclipse TE300, 60x water-immersion CFI Plan-Fluor n.a. 1.2 objective). YFP and rhodamine were excited at 488 and 568 nm, respectively, and fluorescence was collected at 522 (green) and 585 nm (red). ImageJ 1.37v software was used for treatment and analysis of images.

### 2.6. Live Cell Imaging

FAK/YCam cells plated on Matrigel for 2 days were submitted to 100 *μ*M pervanadate treatment in phenol-free EMEM supplemented with 10% FCS and 10 mM HEPES, prior to imaging by confocal microscopy. Z-series stacks (0.35 *μ*m steps) were acquired every 5 minutes for 1 h at 32°C. Representative cells are illustrated from a minimum of 4 independent experiments. Image J software was used to assess the dynamics of FAs and membrane ruffles. Automated counting of FAs in single cells was done after noise removal by thresholding, filtering and applying a size constraint to FAs. Data are presented as mean ± s.e.m. of the number of FAs/cell.

### 2.7. Statistical Analysis

Data were analyzed using Student's *t*-test and differences were considered to be significant at (*) *P* ≤ .05.

## 3. Results

### 3.1. Pervanadate Increases FAK Phosphorylation at FAs

**Figure 1:**
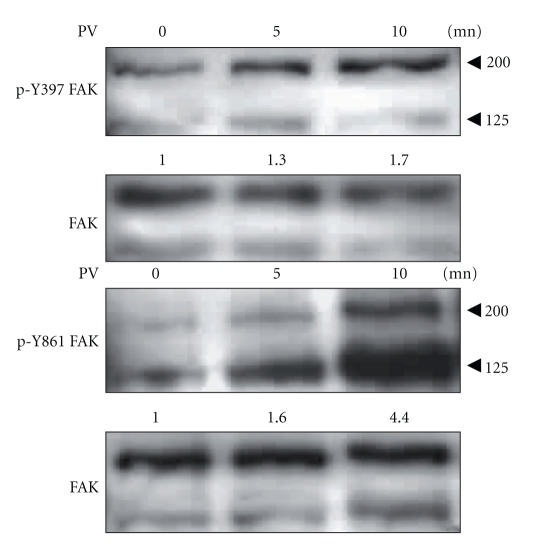
Pervanadate increases FAK phosphorylation at FAs. FAK/Ycam expressed U87-MG cells were treated with 100 *μ*M pervanadate (PV) for the indicated time (minutes). Western blots probed with phospho-specific FAK Abs (against Tyr397 and Tyr861) showed time-dependent increase in phosphorylation. Endogenous FAK was detected at 125 KDa, and exogenous FAK at 200 KDa. Total amount of FAK was verified by stripping and probing with Ab against the Ct domain of FAK. Quantified numbers between blots correspond to the endogenous and exogenous phospho-FAK signal intensities normalized to total FAK. Quantifications were done using Image J software.

Previous studies have shown that upon tyrosine phosphorylation, FAK was excluded from FAs [7]. We have established that FAK is implicated in the disassembly of FAs and that phosphorylation of FAK is the key determinant in the time-residency of FAK at FAs [14]. In order to determine the effect of FAK phosphorylation on its subcellular localization, U87-MG cells stably transfected with FAK/YCam were treated for the indicated times with pervanadate, a phosphatase inhibitor known to increase FAK phosphorylation [7, 15, 16]. Upon pervanadate treatment, phosphorylation at Tyr397 and Tyr861 of FAK/Ycam occurs in a time-dependent manner (Figure 1). Moreover, pervanadate-dependent increased phosphorylation of endogenous FAK is also detected, while the overall amount of FAK remained stable.

### 3.2. Increased Phosphorylated FAK in Membrane Ruffles

**Figure 2:**
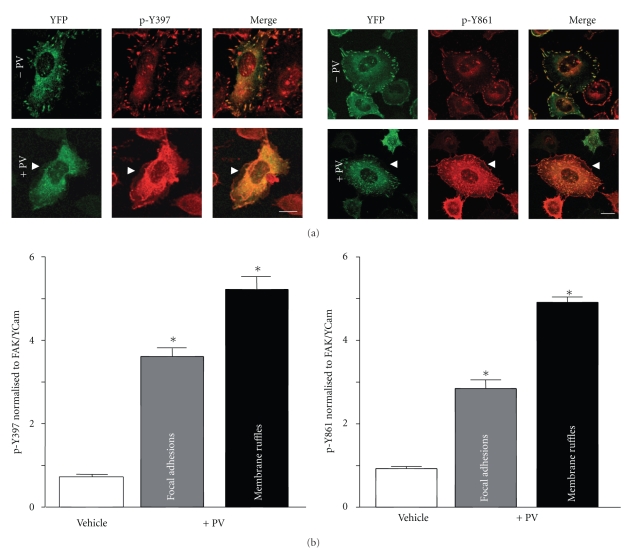
Increased phosphorylated FAK in membrane ruffles upon PV treatment. (a) Cells expressing FAK/Ycam were treated with vehicle or PV for 20 minutes, PFA fixed and immunostained with phospho-specific FAK (Tyr397 and Tyr861) Abs. Note the increase in global fluorescence in PV treated cells. Arrowheads indicate the presence of phospho-FAK in membrane ruffles. Scale bar, 10 *μ*m. (b) Graphs present p-Y397-FAK and p-Y861-FAK intensities relative to total FAK intensity (YFP). Data are means ± s.e.m. from 6 independent experiments.

To characterize the effect of FAK hyperphosphorylation on its subcellular localization, cells grown on Matrigel-coated imaging dishes were incubated with pervanadate. Cells expressing exogenous FAK are identified by emission of the YFP signal. Immunostaining images show a global increase in phosphorylated FAK signal intensity as seen in cells stained for Tyr397 and Tyr861 compared to untreated cells (Figure 2(a)). At FAs, the ratio of phosphorylated FAK over total FAK is increased by 3.6-fold for Tyr397 and 2.9-fold for Tyr861 compared to control cells (Figure 2(b)). However, FAK staining at FAs was more diffuse after PV treatment, suggesting redistribution of FAK upon phosphorylation, as previously described in fibroblasts [7]. As such, phosphorylated FAK appeared to be highly localized at membrane ruffles after pervanadate, with the ratio of phosphorylated FAK over total FAK being increased 5.3-fold for Tyr397 and 4.9-fold for Tyr861 compared to control cells. Of note, the enhanced localization of FAK in membrane ruffles was not as clear in cells visualized by YFP signal. One possible explanation might be that FAK phosphorylation induces cleavage of FAK with the N-terminus of FAK which contains the YFP moiety thus being no longer addressed to FAs. However, immunostaining experiments with Abs against the kinase, Ct or FAT domains of FAK did not reveal more intense FAK staining at membrane ruffles after pervanadate (not shown). Our results suggest that a small fraction of FAK localizes at membrane ruffles that is highly phosphorylated, which is consistent with high FAK activity.

### 3.3. Increase FA Disassembly upon FAK Hyperphosphorylation

**Figure 3:**
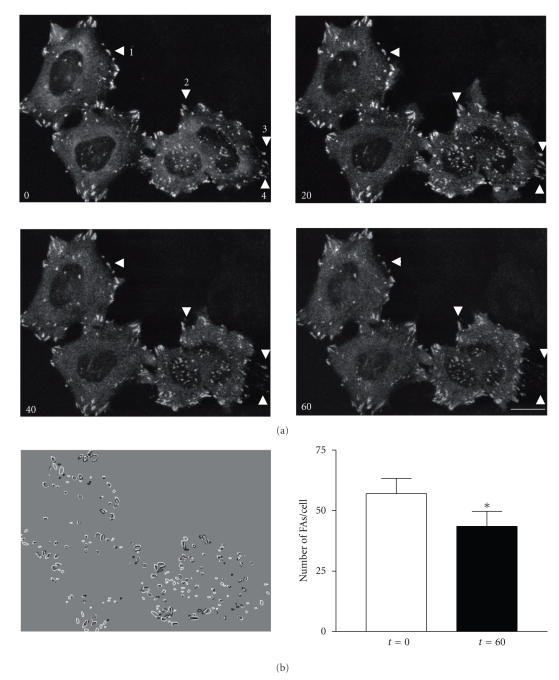
Analysis of FA behavior upon FAK hyperphosphorylation. (a) Confocal time-lapse images of FAK/Ycam expressed cells were collected every 5 minutes for 1 hour after PV addition. Confocal images of living cells at 0, 20, 40, and 60 minutes are shown. Arrowheads, labeled 1 to 4, indicate areas of FA disassembly. Scale bar, 10 *μ*m. (b) Image J software was used to apply a filter and a size constraint to FAs and the image shown results from the merging of images at *t* = 60 (black) and *t* = 0 (white). Newly formed FA (black) and dissociated FA (white). Graph represents the number of FA/cell that was measured by the segmentation/size criteria process both before PV (*t* = 0, white bar) and after 60 minutes PV treatment (*t* = 60, black bar) from 8 cells (mean ± s.e.m.). Note the significant decrease in the number of FAs in cells treated with PV compared to untreated cells (*P* < .05).

To further determine whether the localization of FAK is indeed sensitive to tyrosine phosphorylation, time-lapse imaging on live FAK/Ycam cells was carried out. Cells were treated with pervanadate and observed over a 1 hour period (Figure 3(a)). Several FAs underwent disassembly during pervanadate-induced FAK hyperphosphorylation. Quantification of FA dynamics revealed a decrease in the number of FAs per cell (Figure 3(b)) as well as an overall decrease in the fluorescence intensities of FAs (not shown) after PV treatment, that were sometimes accompanied by clear cellular movements (Figure 3). Out of the 30% of cells analyzed, a complete loss of FAs was observed 1 h after pervanadate. These results suggest that hyperphosphorylation of FAK leads first to the exclusion of FAK from FAs followed by disassembly of FAs.

### 3.4. Decreased Colocalization of FAK to Vinculin after Pervanadate Treatment

**Figure 4:**
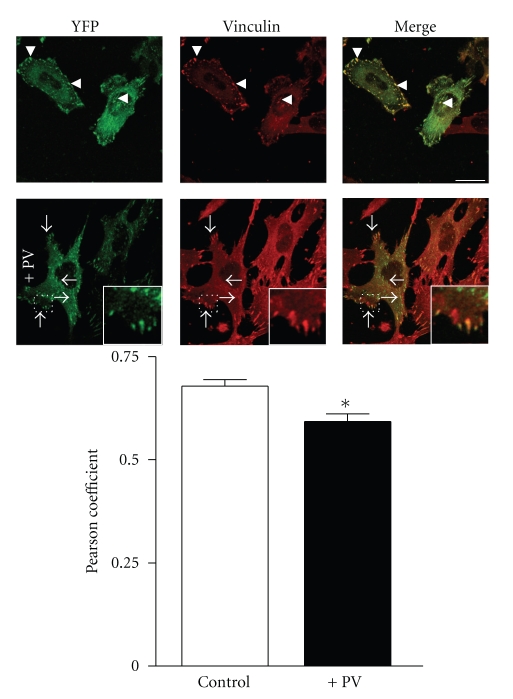
Colocalization of FAK and vinculin. After PV treatment, cells were fixed and immunolabeled for vinculin (Rhodamine Red-X). Arrowheads point to FAs in untreated cells and arrows point to FAs in cells treated for 20 minutes with PV. Inserts are magnified images of indicated boxed regions. Note the reduction in the degree of colocalization of FAK (green) with vinculin (red) at FAs (arrows). Quantification of colocalization was determined by Pearson's coefficient using Image J software and the graph represents means ± s.e.m. of more than 20 FAs analyzed per condition.

In order to test this hypothesis, immunostaining experiments were done on cells treated for 20 minutes with PV and labeled for FAK and vinculin, a marker of FAs. FAK and vinculin completely colocalized at FAs in untreated cells (arrowheads, Figure 4), while some peripheral FAs stained only for vinculin and not FAK in PV treated cells (arrows, Figure 4). Moreover, ventral FAs were marked only for FAK and not vinculin (Figure 4). These observations were confirmed by quantification analysis using Pearson's coefficient that determines the degree of overlap between two paired images (Figure 4). The coefficient is relatively high in control cells (*R* = 0.68 ± 0.01, *n* = 20) but significantly lower in PV-treated cells (*R* = 0.59 ± 0.02, *n* = 22).

### 3.5. Increase FAK at Cell Membrane

**Figure 5:**
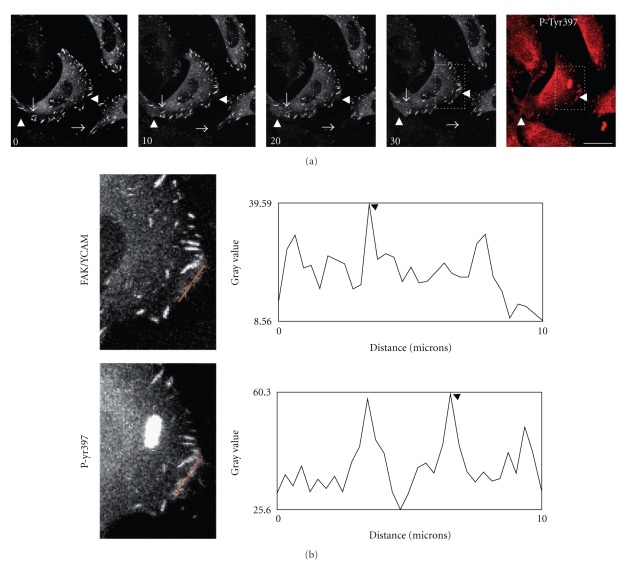
Increased phosphorylated FAK at cell membrane. (a) Images of PV-treated cells were taken by confocal microscopy at 5 minutes intervals for 30 minutes, then cells were fixed and immunolabeled for p-Tyr397-FAK (Rhodamine Red-X). Scale bar, 20 *μ*m. Note membrane ruffle formation (arrowheads) and loss of FAs (arrows) upon addition of PV. Moreover, membrane ruffles show strong P-Tyr397-FAK labeling (right panel, red). (b) Boxed regions in panel (a) are magnified in panel (b) and correspond to FAK/Ycam (green) and p-Tyr397-FAK (red) signals at 30 minutes after PV. Fluorescence intensity analyses were quantified at the membrane region. Fluorescence intensity was measured along the 10 *μ*m line (red) using Plot Profile, Image J software, with grey values ranging from 0 to 256. Note the increase in phospho-FAK signal at the cell membrane compared to total FAK (maximal intensities indicated by black arrowheads).

In order to correlate membrane ruffle formation and localization of hyperphosphorylated FAK, FAK/Ycam cells were incubated with PV and followed by time-lapse imaging. After 30 minutes, cells were fixed, and labeled for phospho-Tyr397-FAK. Of note, while 75% of cells presented no change in membrane ruffle formation in PV-treated cells, the remaining 25% showed FAK disassembly together with membrane ruffle formation (Figure 5(a)). To examine whether newly formed ruffles contained phosphorylated FAK, PV-treated cells were fixed, permeabilized and labeled for phospho-Tyr397-FAK. Results revealed that phospho-Tyr397 is detected at FAs but also at membrane ruffles (Figure 5(b)), indicating that activated FAK was present in membrane ruffles. Intensity analysis of the cell membrane region pointed by an arrowhead showed a significant increase in phosphorylated FAK over total FAK (60.3 and 39.59 maximal fluorescence intensities, resp.).

### 3.6. Src-Dependent Localization of FAK

**Figure 6:**
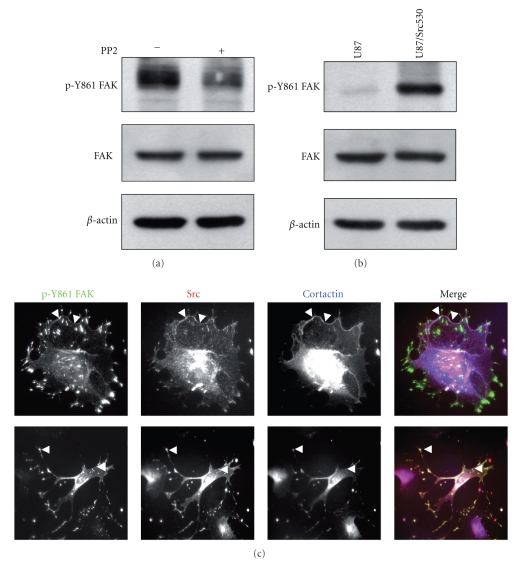
Effects of Src on phosphorylated FAK. (a) Western blots done on U87 cell lysates incubated with or without 10 *μ*M PP2 for 30 minutes and probed for p-Y861-FAK and total FAK. P-Tyr861 decreased after PP2, consistent with Src activity being necessary for Tyr861 phosphorylation. (b) U87 cells transiently expressing Y530F-Src. Blots were probed for p-Y861 FAK and total FAK. Total amounts of proteins in panels (a) and (b) were monitored by stripping and blotting for *β*-actin. (c) Cells expressing transiently Y530F-Src (red) were fixed, immunostained for p-Y861-FAK (green), and cortactin (blue), used as a marker for invadopodia. Arrowheads show regions of colocalization of p-Tyr861-FAK, Src, and cortactin at invadopodia and cell membrane.

Our observations indicate that tyrosine phosphorylation of FAK induces delocalization of FAK from FAs to membrane ruffles since increased phosphorylated FAK was found at membrane ruffles after PV treatment (Figure 2). Phosphorylation of FAK at Tyr397 creates a binding site for Src, which in turn phosphorylates the other tyrosine residues of FAK. It has been reported that Tyr861 is a Src-specific substrate [17, 18]. In agreement, we now report that in human astrocytomas, U87 cells treated with PP2, a selective Src inhibitor, showed a significant decrease in phosphorylation of FAK at Tyr861 (Figure 6(a)). Moreover, transfection of these cells with the constitutively active form of Src, Src-Y530F, results in an increase in FAK-Tyr861 phosphorylation levels (Figure 6(b)). This demonstrated that Tyr861 is a Src-specific target in human astrocytomas and therefore suggests that FAK translocation from FAs to membrane ruffles may be a consequence of Src activity and one of the first steps in oncogenic transformation. Indeed, cellular localization of phosphorylated FAK shows either membrane localization (Figure 6(c) top panels) comparable with that observed after pervanadate treatment or invadopodia localization (Figure 6(c) bottom panels). The invadopodia are characterized by localization of the specific marker cortactin (Figure 6(c)). This difference in localization may depend on the stage of oncogenic transformation.

## 4. Discussion

Tyrosine phosphorylation is clearly implicated in the turnover of FAs, yet the molecular mechanism underlying this process is still obscure. We previously described a rapid flux of FAK between FAs and cytosol [13]. Increasing FAK phosphorylation led to increased time-residency of FAK at FAs, whereas decreasing phosphorylation gave rise to a decreased time-residency [14]. Macroscopically, the consequence of FAK hypophosphorylation is a decrease in global FA turnover as reported in several studies using Y397F-FAK [6, 14, 19]. Src family kinases have been implicated in phosphorylation-induced exclusion of FAK from FAs [7], but the relationships between FAK phosphorylation, FAK trafficking and FA turnover have never been described. In this study, we analyzed FAK trafficking upon FAK phosphorylation and demonstrated that phosphorylated FAK was translocated from FAs to membrane ruffles via presumably a Src-dependent mechanism, leading in turn to FA disassembly and formation of membrane ruffles. This process links the effects of FAK in promoting integrin-stimulated cell motility to the implication of FAK in cell invasion [20, 21].

Coordinated disassembly of FAs is known to be required for efficient cell migration, yet the process of FA disassembly is only poorly understood. The relative lack of information reflects that FA disassembly and assembly occur simultaneously, and until recently, model systems in which disassembly is kinetically separated from assembly were unavailable. To circumvent this, recent studies have employed a method allowing coordination of FA disassembly [14, 19]. The strategy is based on earlier observations that showed growing microtubules transiently contacting FAs and inducing their disassembly [22]. Nevertheless, despite the clear insights provided by the “nocodazole wash-out” model [22] in the mechanism of FA disassembly, some important pathways were apparently not required. For example, Src family kinases were found not to be essential in microtubule-induced FA disassembly [19] contrarily to what has been demonstrated for adhesion turnover in migrating fibroblasts [6, 12] or colon carcinoma cells [23]. Therefore, we used another strategy based on FAK phosphorylation in order to better visualize and analyze FA disassembly. Indeed, FAK phosphorylation appears to be a major and obligatory step in this process [12–14, 19, 21, 24]. After pervanadate, FAK is hyperphosphorylated [7, 15], leading to an increased cell ratio content of phosphorylated FAK over FAK, and thereby allowing analysis of the cellular localization of phosphorylated FAK. Indeed, our immunofluorescence experiments in pervanadate-treated cells reveal a membrane localization of phosphorylated FAK that was hardly detected by visualizing the YFP moiety of the FAK/YCam chimera (Figure 2(a)). Upon pervanadate treatment, FAK is first delocalized from FAs as demonstrated with colocalization analysis of FAK and vinculin and after the phosphorylation process. This is consistent with the gradual disassembly of FA observed upon pervanadate treatment (Figure 5). Hence, these data provide strong, yet indirect, evidence for molecular changes at FAs, starting with high FAK phosphorylation and followed by FA disassembly and p-FAK trafficking to membrane ruffles.

The question now is to explain what might be the biological significance of high phosphorylated FAK at membranes ruffles. Recently, Tyr397, the autophosphorylation site of FAK was shown to be essential for the spatial organization of the leading edge of migrating cells [25]. FAK and its phosphorylation process has also been shown to be necessary for lamellipodial formation and progression [26, 27] and tyrosine phosphorylation of FAK has been associated with lamellipodial progression in human neuroblastoma cells. Moreover, stable localization of FAK at FAs has been observed in migrating fibroblasts, as well as transient recruitment of FAK to lamellipodial extensions with formation of a signaling complex including FAK, Src, p130Cas and Dock 180, involved in cell invasion [20]. Therefore, phosphorylated FAK at membrane ruffles might be one of the early steps in the process of oncogenic transformation. Indeed, when U87 cells are transfected with the constitutive active form of Src, these cells displayed increased level of phosphorylated FAK at tyr861, and a transformed phenotype characterized by the presence of podosomes as already described [28]. Because of the presence of markers of FAK activation in podosomes [29], we believe that upon Src activation, early changes in the cell phenotype involve loss of focal adhesions and ruffle formation, and subsequently, formation of podosomes. Accordingly, using a temperature-sensitive mutant of Src, actin rearrangement has been followed upon switch from the restrictive to the permissive temperature [30]. In this study, formation of Src-containing membrane ruffles was evident 16 hours after the switch.

In conclusion, in response to migration- and invasion-associated stimuli, high levels of Src expression and activity in tumor cells allows for a positive FAK-Src feedback loop leading to subsequent phosphorylation of FAK. Phosphorylated FAK shuttles rapidly from FAs to membrane ruffles, which may be one way for cells to enhance highly dynamic turnover of FAs and to relocalize adhesion proteins into nascent adhesions formed at the lamellipodia. Our findings provide new insights into the molecular mechanism involving FAK phosphorylation in promoting tumor cell migration. Thus, targeting of FAK phosphorylation may be a strategical basis for cancer treatment. Recently, several small molecular inhibitors have been developed that target specifically the Y397 autophosphorylation of FAK, such as TAE226, a compound that shows effective inhibition of angiogenesis, and tumor growth when treated in combination with imatinib [31, 32]. The promising inhibitor Y15 specifically blocks FAK autophosphorylation and thus its overall phosphorylation, leading to cell detachment and pancreatic tumor regression in vivo [33, 34].

## Abbreviation

Ab: antibody
YFP: yellow fluorescent protein
FA: focal adhesion
FAK: focal adhesion kinase
PV: pervanadate
YCam: yellow cameleon.
